# Biochemical investigations of the mechanism of action of small molecules ZL006 and IC87201 as potential inhibitors of the nNOS-PDZ/PSD-95-PDZ interactions

**DOI:** 10.1038/srep12157

**Published:** 2015-07-16

**Authors:** Anders Bach, Søren W. Pedersen, Liam A. Dorr, Gary Vallon, Isabelle Ripoche, Sylvie Ducki, Lu-Yun Lian

**Affiliations:** 1 Department of Drug Design and Pharmacology, Faculty of Health and Medical Sciences, University of Copenhagen, Universitetsparken 2, DK-2100 Copenhagen, Denmark; 2 NMR Centre for Structural Biology, University of Liverpool, UK L69 7ZB Liverpool; 3 Clermont Université, ENSCCF, CNRS UMR 6296, Institut de Chimie de Clermont-Ferrand, BP10187, F-63174 Aubière, France

## Abstract

ZL006 and IC87201 have been presented as efficient inhibitors of the nNOS/PSD-95 protein-protein interaction and shown great promise in cellular experiments and animal models of ischemic stroke and pain. Here, we investigate the proposed mechanism of action of ZL006 and IC87201 using biochemical and biophysical methods, such as fluorescence polarization (FP), isothermal titration calorimetry (ITC), and ^1^H-^15^N HSQC NMR. Our data show that under the applied *in vitro* conditions, ZL006 and IC87201 do not interact with the PDZ domains of nNOS or PSD-95, nor inhibit the nNOS-PDZ/PSD-95-PDZ interface by interacting with the β-finger of nNOS-PDZ. Our findings have implications for further medicinal chemistry efforts of ZL006, IC87201 and analogues, and challenge the general and widespread view on their mechanism of action.

Neuronal nitric oxide synthase (nNOS) is a nitric oxide (NO)-producing enzyme found in neuronal synapses where it interacts with the scaffolding protein postsynaptic density protein-95 (PSD-95). PSD-95 connects to *N*-methyl-d-aspartate (NMDA) receptors and thereby forms the ternary nNOS/PSD-95/NMDA receptor complex. This ternary complex ascertains efficient and localized NO production as a result of glutamate-mediated Ca^2+^ influx via the NMDA receptor (Fig. 1a)^1^,^2^. In disease-states involving glutamate-mediated excitotoxicity, such as ischemic stroke, neuropathic pain and neurodegenerative diseases, excessive NMDA receptor activation leads to harmful production of NO and hence neuronal death^3^,^4^,^5^. NMDA receptor antagonists have failed clinical trials due to, among other reasons, severe side effects^6^,^7^,^8^, but PSD-95 inhibitors such as the monomeric peptide Tat-NR2B9c^5^,^9^,^10^ or the dimeric peptide-analogue Tat-*N*-dimer^11^ appear more promising. These compounds bind the postsynaptic density protein-95/disks large/zonula occludens (PDZ) domains PDZ1 and PDZ2 of PSD-95 and uncouple the harmful production of NO from Ca^2+^ influx without disturbing NMDA receptor function. Therefore, PSD-95 inhibitors are believed to provide a safer mechanism of action than NMDA receptor antagonists. However, PSD-95 has many endogenous interaction partners and plays a key role in organizing protein networks and receptor complexes in neurons. Prolonged treatment with PSD-95 inhibitors, e.g. in relation to pain, could therefore be associated with side effects. Instead, compounds that specifically inhibit the nNOS/PSD-95 interaction without disturbing other pivotal PSD-95-mediated interactions (Fig. 1a) could provide promising alternatives^12^.

PDZ domains are small (~90 amino acids) and globular in shape. They are highly abundant in eukaryotic cells and often found in scaffolding proteins, where they mediate the formation of protein signalling networks^13^,^14^. PDZ domains usually interact with the C-terminus end (~4–7 amino acids) of the interaction partner, and the specificity of PDZ domains has been well-characterized by studying affinities to smaller linear peptides derived from natural binding partners^15^,^16^. In this canonical PDZ-ligand binding mode, the C-terminal peptide fits into a groove formed by the β2 strand and α2 helix of the PDZ domain. The amide backbone of the peptide ligand interacts via pivotal hydrogen bonds with the conserved ‘GLGF’ loop and β2-strand of the PDZ domain^14^,^17^,^18^,^19^. The binding pockets of PDZ domains are shallow and narrow, thus it has not been possible to identify small molecules with submicromolar affinities to any PDZ domain; however, several potent peptidomimetic structures have been identified^14^.

In addition to forming canonical interactions with C-terminal peptide parts, PDZ domains can recognize internal motifs^20^,^21^,^22^. The interaction between nNOS and PSD-95 is mediated by such a non-canonical PDZ-PDZ domain interaction, where the so-called β-finger of the extended nNOS-PDZ domain (Fig. 1b) binds to the peptide-binding pockets of the PDZ1 or PDZ2 domain of PSD-95^22^. A similar binding mode is seen in the nNOS/Syntrophin interaction^20^,^23^. Interestingly, mutation of Arg121 positioned at the β-finger to an uncharged amino acid residue has shown to destabilize the β-finger portion of nNOS-PDZ leading to loss of its structure, by which nNOS can no longer interact with PSD-95-PDZ2^22^. Thus, the salt bridge between Asp62 on the canonical part of nNOS-PDZ and Arg121 of the nNOS β-finger (Fig. 1b) is essential in stabilizing the β-finger and hence facilitating nNOS interaction with PSD-95.

ZL006 (Fig. 1c) has been shown to inhibit the nNOS/PSD-95 interaction in co-immunoprecipitation assays of extracts from glutamate- or ischemia-induced cultured neurons and cortical brain^24^. The canonical PDZ-mediated interactions nNOS/CAPON (carboxy-terminal PDZ ligand of nNOS), PSD-95/SynGAP (synaptic GTPase activating protein), and PSD-95/GluN2B were not inhibited by ZL006 under these conditions. Also, ZL006 did not affect the NMDA receptor function (ion flux), nNOS expression or nNOS catalytic activity, indicating a specific action on the nNOS/PSD-95 complex^24^. ZL006 inhibited NMDA receptor-mediated NO synthesis, and showed robust neuroprotective properties in neurons and animal ischemic stroke models,^24^ but not when the nNOS gene was knocked out.^24^ ZL006 was described as being specifically *de novo* designed to create a compound that interacts with and disrupts the Asp62-Arg121 salt bridge via its carboxylic acid group. The molecule was predicted to engage with hydrophobic residues (Leu107 and Phe111) on nNOS-PDZ, thereby preventing a conformational change essential for formation of the nNOS-PDZ/PSD-95-PDZ complex^24^,^25^. IC87201 (Fig. 1c) was found by screening and shown to disrupt the nNOS/PSD-95 interaction in an *in vitro* assay where biotin-labelled PSD-95-PDZ1-3 was captured by immobilized nNOS (recombinant nNOS1-299) followed by streptavidin-europium-mediated detection^26^. IC87201 did not affect the PSD-95/cypin-interaction or any of 34 targets in a panel of receptors, ion channels, and transporters. Also, the compound blocked NMDA-induced 3′,5′-cyclic guanosine monophosphate (cGMP) production in hippocampal cultures, and showed analgesic properties in two mice pain models^26^.

ZL006 and IC87201 are being described in literature as efficient inhibitors of the nNOS/PSD-95 interaction^12^,^24^,^25^,^26^,^27^,^28^,^29^,^30^,^31^, and have shown great effects in animal models of ischemic stroke^24^, pain^26^, depression^27^, and recently, regenerative repair after stroke^30^. For ZL006, the proposed mechanism for this is a direct binding of ZL006 to the extended nNOS-PDZ domain at the β-finger, thus preventing interaction to PSD-95^24^. This hypothesis has not been corroborated with detailed molecular or biostructural evidence, but has nevertheless become the prevailing explanation for the pharmacological effects of ZL006^12^,^24^,^25^,^27^,^28^. Also, ZL006 and IC87201 are structurally very similar, and, therefore, the mechanism of IC87201 is assumed to be the same as suggested for ZL006^12^,^27^,^28^,^29^,^31^. Because this proposed mechanism is both intriguing and potentially relevant for future drug discovery efforts, we decided to examine it further with techniques not previously applied for either ZL006 or IC87201, namely fluorescence polarization (FP), isothermal titration calorimetry (ITC) and ^1^H-^15^N HSQC NMR. These methods are known to be very reliable for investigating ligand-protein interactions. We hoped to provide evidence for the reported inhibitory properties of the nNOS-PDZ/PSD-95-PDZ interaction via interaction with the β-finger, enabling us to explore these compounds further using medicinal chemistry approaches. However, our results robustly demonstrate that neither ZL006 nor IC87201 directly interacts with extended nNOS-PDZ or any of the PSD-95 PDZ domains *in vitro*. Neither do they inhibit the formation of the nNOS-PDZ/PSD-95-PDZ complexes. Therefore, we believe the correct mechanism of action of ZL006 and IC87201 has yet to be revealed, but hopefully our findings will stimulate further investigations into these biological active and efficient compounds.

## Results

### Fluorescence Polarization Assay

**Figure 2:**
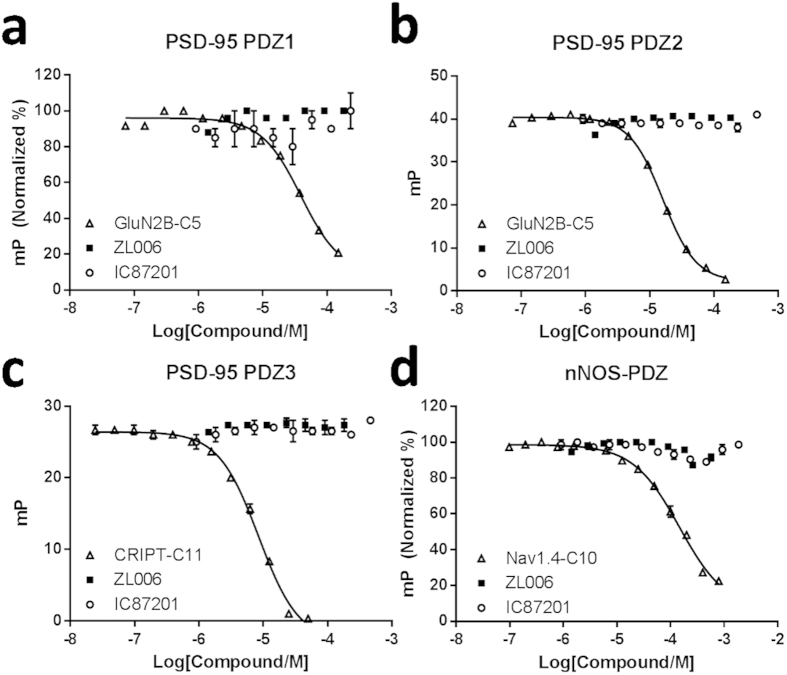
ZL006 and IC87201 tested for affinities to PDZ1, PDZ2, PDZ3 of PSD-95 and nNOS-PDZ. (**a**) Cy5-GluN2B as probe (50 nM), [PDZ1] = 10 μM (ZL006) or 18 μM (IC87201). (**b**) Cy5-GluN2B as probe (50 nM), [PDZ2] = 6 μM. (**c**) Cy5-CRIPT as probe (50 nM), [PDZ3] = 5 μM. (**d**) Cy5-Nav1.4 as probe (50 nM, *K*_d_ = 20 ± 1 μM), [nNOS-PDZ] = 20 μM (ZL006) or 10 μM (IC87201), Nav1.4-C10: *K*_i_ = 45 ± 4 μM. Error bars indicate SEM based on three individual measurements.

ZL006 and IC87201 were expected to bind nNOS-PDZ at its β-finger. However, to ascertain that the published biochemical results could not be explained by inhibition of canonical PDZ domain-mediated interactions, we checked the potential affinities of ZL006 and IC87201 towards PDZ1, PDZ2, PDZ3 of PSD-95 and nNOS-PDZ in our well-established FP assay^16^,^32^,^33^. Fluorescent probes were made by attaching fluorophores (Cy5) to C-terminal 10- or 11-mer (C10 or C11) peptide ligands representing canonical and natural occurring protein partners (Supplementary Table S1). Saturation curves were then created by applying increasing concentrations of PDZ (typically 0–150 μM) to a low (5–50 nM) and fixed concentration of probe, and *K*_d_ values between probe and PDZ were determined^16^,^32^,^33^. The affinities for both ZL006 and IC87201 towards the PDZ domains were investigated in an inhibition FP assay using constant concentrations of probe and PDZ and increasing concentrations of test compound or inhibitory control peptide (Supplementary Table S1). The results clearly showed that ZL006 and IC87201 at concentrations reaching 500–1800 μM did not inhibit any of the probe-PDZ interactions involving PDZ1, PDZ2, PDZ3 of PSD-95 or nNOS-PDZ, while all control peptides demonstrated expected potencies^16^,^32^ (Fig. 2). Thus ZL006 and IC87201 do not bind the canonical PDZ ligand binding sites.

**Figure 3:**
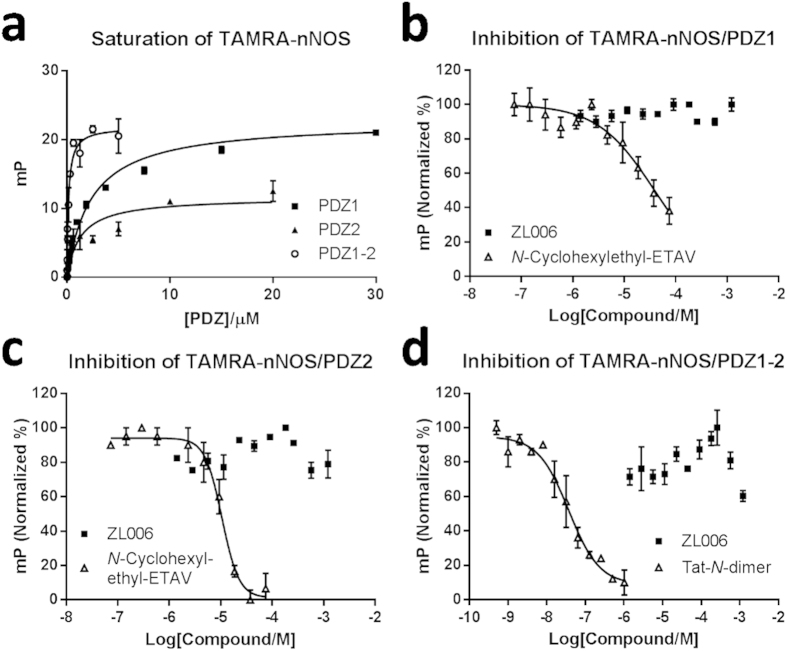
ZL006 and IC87201 tested in the ‘direct’ FP assay for their ability to inhibit the interactions between TAMRA-nNOS and PDZ1, PDZ2, or PDZ1-2 of PSD-95. (**a**) Saturation curves between TAMRA-nNOS (5 nM) and PDZ1, PDZ2, and PDZ1-2 of PSD-95. *K*_d_ values: 2.4 ± 0.4 μM (PDZ1), 1.1 ± 0.3 μM (PDZ2), and 0.15 ± 0.03 μM (PDZ1-2). (**b**) TAMRA-nNOS (5 nM)/PDZ1 (7.5 μM) inhibited by *N*-Cyclohexylethyl-ETAV (*K*_i_ ~ 3.9 μM) and ZL006. (**c**) TAMRA-nNOS (5 nM)/PDZ2 (2.5 μM) inhibited by *N*-Cyclohexylethyl-ETAV (*K*_i_ = 2.0 ± 0.5 μM) and ZL006. (**d**) TAMRA-nNOS (5 nM)/PDZ1-2 (0.3 μM) inhibited by Tat-*N*-dimer (IC_50_ = 40 ± 15 nM) and ZL006. Error bars indicate SEM based on three individual measurements.

Next, we designed a novel ‘direct’ FP assay in order to measure if ZL006 and IC87201 could inhibit the interaction between nNOS-PDZ and PSD-95 PDZ domains (PDZ1, PDZ2, PDZ1-2). Inhibitory effects in this assay would support the proposed mechanism of ZL006 and IC87201 binding to the β-finger of nNOS-PDZ and allosterically inhibit the nNOS-PDZ/PSD-95-PDZ interactions. The assay was established by creating a fluorescent version of nNOS-PDZ by TAMRA-labelling nNOS-PDZ via an introduced cysteine residue (V36C). *K*_d_ values for PDZ1 and PDZ2 towards this TAMRA-nNOS were measured by saturation curves to be 2.4 μM and 1.1 μM, respectively (Fig. 3a), which correlate with the literature^20^,^34^. PDZ1-2 was found to interact with TAMRA-nNOS with high affinity (*K*_d_ = 0.15 ± 0.03 μM) (Fig. 3a); while TAMRA-nNOS and PDZ3 did not bind each other (data not shown), as also reported by others^34^. We then tested ZL006 and IC87201 for their ability to inhibit the TAMRA-nNOS/PSD-95-PDZ interactions. ZL006 revealed no activity in this assay involving any of the PSD-95 PDZ domains when tested at up to 1200 μM (Fig. 3b–d). This demonstrates that ZL006 does not inhibit the nNOS-PDZ/PSD-95-PDZ interaction, and indicates that ZL006 does not perturb the nNOS β-finger. Control compounds, *N*-cyclohexylethyl-ETAV^16^,^32^ and Tat-*N*-dimer^11^, clearly inhibited the interaction as expected, as they bind the canonical PDZ pockets of PDZ1 and PDZ2 and displace TAMRA-nNOS (Fig. 3b–d). Unfortunately, IC87201 showed high degree of fluorescence-based artefactual signal when using TAMRA-nNOS as probe. This is a common problem for fluorescence-based assays in relation to small molecules^35^,^36^, and it prevented reliable testing of IC87201 above 7.3 μM. However, up to 7.3 μM no inhibition of TAMRA-nNOS/PSD-95-PDZ interactions could be measured (data not shown).

**Figure 4:**
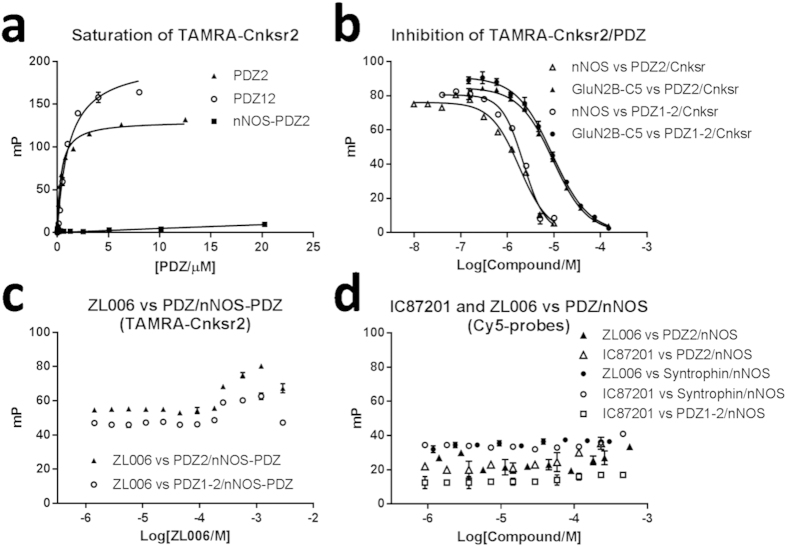
ZL006 and IC87201 were tested in the ‘indirect’ FP assay for their ability to inhibit the interactions between nNOS-PDZ and PDZ domains from PSD-95 and α1-Syntrophin. (**a**) Saturation of TAMRA-Cnskr2 (5 nM) with PSD-95-PDZ2 (*K*_d_ = 0.38 ± 0.03 μM), PSD-95-PDZ1-2 (*K*_d_ = 1.1 ± 0.1 μM) and nNOS-PDZ (*K*_d_ > 190 μM). (**b**) TAMRA-Cnskr2 (5 nM)/PDZ2 (1.5 μM) inhibited with nNOS-PDZ (*K*_i_ = 88 ± 14 nM) and pentapeptide GluN2B-C5 (*K*_i_ = 1.6 ± 0.02 μM); and TAMRA-Cnskr2 (5 nM)/PDZ1-2 (1 μM) inhibited with nNOS-PDZ (*K*_i_ = 810 ± 23 nM) and GluN2B-C5 (*K*_i_ = 4.8 ± 0.3 μM). (**c**) ZL006 (0-2900 μM) tested against TAMRA-Cnskr2 (5 nM)/PSD-95-PDZ2 (1.5 μM)/nNOS-PDZ (1 μM) and TAMRA-Cnskr2 (5 nM)/PSD-95-PDZ1-2 (1 μM)/nNOS-PDZ (2.5 μM). The minor increases in mP at high concentrations, 260-2900 μM, were due to fluorescence artefacts. (**d**) ZL006 and IC87201 tested against Cy5-Cnskr2 (5 nM)/PSD-95-PDZ2 (3 μM)/nNOS-PDZ (5 μM), and against Cy5-Sapk3 (5 nM)/α-Syntrophin-PDZ (14 μM)/nNOS-PDZ (10 μM); and IC87201 tested against Cy5-Cnskr2 (5 nM)/PSD-95-PDZ1-2 (1 μM)/nNOS-PDZ (5 μM).

In a final attempt to provide evidence for the expected mechanism of ZL006 and IC87201 using FP, we designed an ‘indirect FP assay’ measuring the abilities of the compounds to inhibit the nNOS-PDZ/PSD-95-PDZ interaction in the presence of a PSD-95-PDZ-binding probe. First we tested a series of 23 fluorescent-labelled C-terminal peptide ligands (Supplementary Table S1) derived from PDZ-interacting proteins in order to find a probe with good activity towards the PSD-95 PDZ domains and no or little activity against nNOS-PDZ (Supplementary Fig. S1). The Cnskr2 probe fulfilled these selectivity criteria and was used to generate saturation curves with PDZ2 and PDZ1-2 of PSD-95 (Fig. 4a). The Cnskr2/PDZ interactions were subsequently inhibited with unlabelled nNOS-PDZ or the control peptide GluN2B-C5 representing the C-terminal pentapeptide of the NMDA receptor subunit GluN2B, and it was seen that nNOS-PDZ inhibited the Cnskr2/PDZ interactions potently demonstrating its high affinity to both PDZ2 and PDZ1-2 of PSD-95 (Fig. 4b). Next, fixed concentrations of TAMRA-Cnskr2, PSD-95-PDZ2/1-2, and nNOS-PDZ were applied, corresponding to the conditions at IC_50_ of the nNOS-PDZ inhibition curve (Fig. 4b) where sensitivity of the assay is greatest. If the compounds inhibited the nNOS-PDZ β-finger-mediated interaction to PDZ2 or PDZ1-2 of PSD-95, they would liberate PSD-95 PDZ domains from nNOS-PDZ thereby enabling PSD-95 PDZ domains to interact with the peptide probe (TAMRA-Cnskr2) resulting in a rise in FP signal. However, when testing ZL006 under these conditions we observed no rise in FP signal; only a flat line was generated (Fig. 4c). This indicates, again, that ZL006 is not able to inhibit nNOS-PDZ/PSD-95-PDZ interactions. Finally, we established the assay using Cy5-probes instead of TAMRA-probes, in order to diminish artefactual FP signal when testing IC87201. In addition, we investigated if the compounds inhibited the α1-Syntrophin-PDZ/nNOS-PDZ interaction, also mediated by the nNOS β-finger. Here, we used the Sapk3-peptide as it has the desired high affinity to α1-Syntrophin-PDZ and low affinity to nNOS-PDZ (Supplementary Fig. S1). However, also under these conditions we observed no activity of ZL006 or IC87201 when tested up to 500–600 μM (Fig. 4d). Overall, our results indicate that neither ZL006 nor IC87201 inhibit the nNOS-PDZ β-finger-mediated interactions to PDZ domains of PSD-95 and α-Syntrophin.

### Isothermal Titration Calorimetry

**Figure 5:**
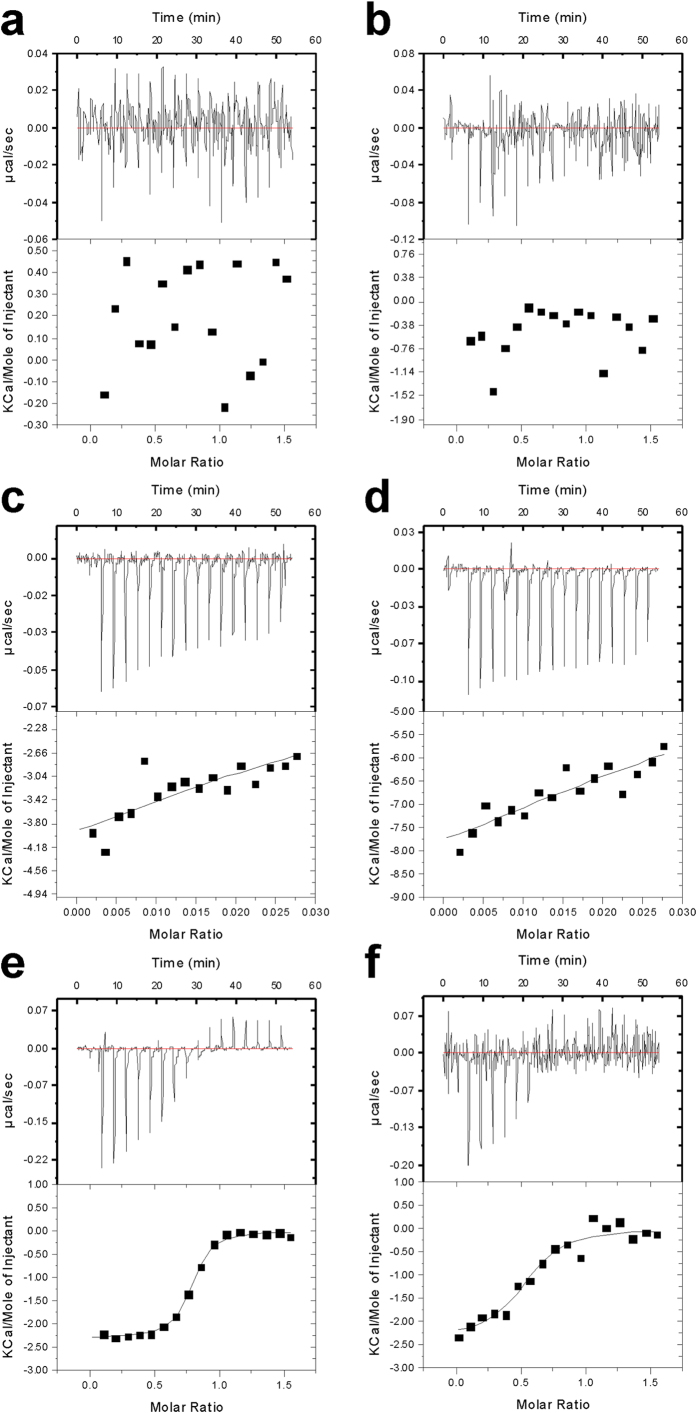
ITC curves. Plots showing titrations into PDZ domains fitted to a one-site model. IC87201 titration into (**a**) extended nNOS-PDZ and (**b**) PSD-95-PDZ2 shows no binding to either protein. ZL006 titration into (**c**) extended nNOS-PDZ and (**d**) PSD-95-PDZ2 also shows very little binding. (**e**) nNOS-PDZ titrated into PSD-95-PDZ2 shows that PSD-95-PDZ2 binds nNOS-PDZ with N (stoichiometry ratio) = 1.0, *K*_d_ ~ 0.67 μM, ∆H = ∼ −1.7 kcal/mol and T∆S = ∼6.7 kcal/mol. (**f**) As (**e**) but with 1 mM IC87201.

Unlike the FP experiments above, which rely on inhibition of interaction partners, the ITC method is a direct measure of binding, regardless of where it takes place on the protein. We, therefore, titrated ZL006 and IC87201 into solutions of either nNOS-PDZ or PSD-95-PDZ2 to detect heat changes in order to follow potential specific ligand-protein interactions. The data conclusively showed that IC87201 does not bind either the extended nNOS-PDZ or PSD-95-PDZ2 (Fig. 5a-b). Titration of ZL006 into these proteins gave some heat change but the binding was too weak to produce a binding isotherm that would yield a reliable *K*_d_ value (Fig. 5c-d). An experiment performed for the binding between extended nNOS-PDZ and PSD-95-PDZ2 showed that these two proteins bind with *K*_d_ ~ 0.67 μM (Fig. 5e). In the presence of 1 mM IC87201, the affinity between nNOS-PDZ and PSD-95-PDZ2 appeared to be affected although the compound also caused significant interference with the data, reducing its reliability (Fig. 5f). Similar experiments to investigate if ZL006 decreases the affinity between the two PDZ domains also suffered from interference and, hence, deemed unreliable for further interpretation. Overall, ITC indicated that ZL006 and IC87201 do not interact directly or specifically with nNOS-PDZ or PSD-95-PDZ2, nor do they drastically disrupt the complexes once they are formed.

### Nuclear Magnetic Resonance

**Figure 6:**
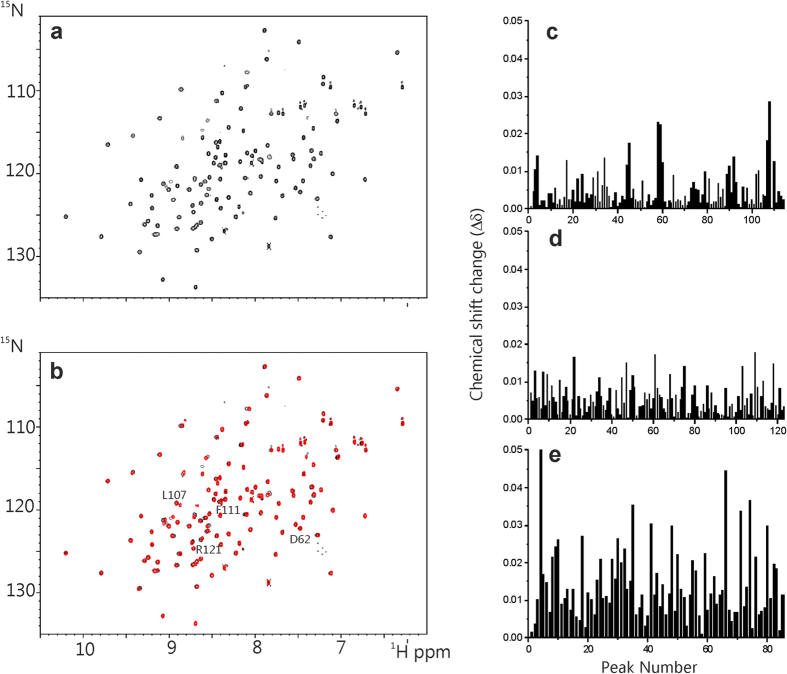
^1^H-^15^N HSQC NMR. (**a**) ^1^H-^15^N of nNOS-PDZ. (**b**) Superimposed ^1^H-^15^N HSQC spectra of ^15^N-labelled nNOS-PDZ in the absence (black) and presence of 20 equivalents of IC87201 (red). Residues Asp62, Leu107, Phe111 and Arg121 are indicated in the spectrum (BMRB Accession Number 4304 ). (**c**–**e**) Histograms of the chemical shift perturbations of (**c**) ^15^N nNOS-PDZ in the presence of 20 equivalents of IC87201, (**d**) ^15^N nNOS-PDZ complexed with unlabelled PSD95-PDZ2 in the presence of 20 equivalents of IC87201, and (**e**) ^15^N PSD-95-PDZ2 complexed with unlabelled nNOS-PDZ in the presence of 20 equivalents of ZL006. Histograms of the chemical shift perturbations were calculated using Δδ = [(ΔH)^2^ + (0.15ΔN)^2^]^1/2^ where ΔH and ΔN are, respectively, the protein and nitrogen chemical shift changes. The presence of even very weak binding would generally cause Δδ > 0.1^37^ in these PDZ domain resonances; the data in (**c**–**e**) show shift perturbations that are much smaller than this. The numbering on the x-axis are peak rather than residue numbers (since the complete backbone resonance assignments are not available). Concentrations of PSD-95-PDZ2 and nNOS-PDZ were between 0.05 mM and 0.250 mM with the concentrations IC87201 and ZL006 adjusted to achieve the stated molar equivalents.

^1^H-^15^N HSQC NMR is another direct method for detecting ligand-protein interactions. However, when we tested IC87201 for binding to the extended nNOS-PDZ (Fig. 6a) only very modest chemical shift changes were observed (Fig. 6b-c), none of which appear to be from residues Asp62, Leu107, Phe111 and Arg121, which are residues proposed to bind or to be affected by ligand-binding^24^,^25^. Typically, chemical shift perturbations of >0.1 ppm and up to 0.3 ppm are observed on binding even very weak ligands with K_d_ ~ 300–500 μM^37^. Here, the maximum shift changes observed were less than 0.05 and are, therefore, considered non-specific. The data for ZL006 were not conclusive since ZL006 caused extensive non-specific shift changes and line-broadening to the ^1^H-^15^N HSQC NMR spectrum of nNOS-PDZ, possibly due to aggregation effects of ZL006. Interestingly, a 20-fold excess of either IC87201 or ZL006 does not appear to disrupt the preformed nNOS-PDZ/PSD95-PDZ2 complex as no shifts changes were observed in the ^1^H-^15^N HSQC spectrum of the complex upon addition of either compound (Fig. 6d,e). Thus, these NMR data are consistent with the ITC experiments and provide further evidence that neither IC87201 nor ZL006 interacts specifically with nNOS-PDZ.

## Discussion

ZL006 and IC87201 have been suggested to target the extended PDZ domain of nNOS and thereby serve as inhibitors of the nNOS/PSD-95 interaction. These compounds would be of great value as pharmacological tool compounds and could represent novel drug leads if they specifically inhibit the nNOS/PSD-95 interaction without affecting other PSD-95-mediated interactions. Indeed ZL006 and IC87201 seem very convincing in neuronal and animal models of ischemic stroke, pain and depression. However, our data – based on sensitive, informative and direct methods, such as FP, ITC, and NMR – demonstrate that ZL006 and IC87201 do not bind the extended nNOS-PDZ domain (or the PSD-95-PDZ domains) and are not able to inhibit nNOS-PDZ/PSD-95-PDZ interactions.

In this study, we have focused on the PDZ domain-mediated interactions of nNOS and PSD-95, while the reported inhibition by ZL006 and IC87201 were based on studies involving full-length or larger protein constructs^24^,^26^,^30^. Our data, therefore, strongly indicate that the true mechanism of action of ZL006 and IC87201 is not via direct binding to the extended nNOS-PDZ domain as originally suggested for ZL006^24^,^25^ and as widely believed for both ZL006 and IC87201^12^,^27^,^28^,^29^,^31^. Given their reported potency, we speculate that ZL006 and IC87201 could bind to other parts of the large and complex 321 kDa homodimer nNOS protein^38^, or perhaps even to other proteins affecting the nNOS/PSD-95 system. In addition, it must be borne in mind that small molecules can affect and disturb biochemical assays in a number of subversive and unforeseeable ways^39^. Specifically, compounds comprising a 2-hydroxybenzylamine moiety, as seen in ZL006 and IC87201 and which is essential for ZL006 activity^24^, are known as phenolic Mannich bases. Such compounds have been identified as ‘frequent hitters’ in AlphaScreening campaigns and can cause artefacts by chelating metal ions or forming quinone methide intermediates that react covalently with protein^40^,^41^. The assays used in literature for demonstrating nNOS/PSD-95 inhibition were based on either co-immunoprecipitation (ZL006)^24^ or capturing assays (IC87201)^26^. These multi-component methods involving antibodies, recognition-tags, complex buffers and surface-immobilization may be prone to pick up compounds with undesirable off-target effects.

In conclusion, the potential of artefactual effects together with our experimental findings using sensitive *in vitro* assays highlight the critical need for further mechanistic studies of ZL006 and IC87201. This is essential to clarify the true promise of these two compounds as pharmacological tools and drug leads and to aid further medicinal chemistry efforts in this important area of neuroscience.

## Methods

### Chemistry

^1^H and ^13^C NMR spectra were recorded on a Bruker Advanced 400 Spectrometer at 400 and 101 MHz, respectively. Chemical shifts (δ) are reported in ppm relative to residual solvent and all *J* values are given in Hz. The following abbreviations are used: singlet (s), doublet (d). High Resolution Electro-Spray Ionisation Mass Spectrum (HR-ESI-MS) of IC87201 was recorded on a Waters/Micromass spectrometer (Micromass, Manchester, UK) at the CRMP (Centre Regional de Mesures Physiques, Clermont-Ferrand, France). Preparative HPLC was performed for peptide-based compounds using an Agilent 1200 system using a C18 reverse phase column (Zorbax 300 SB-C18, 21.2 mm × 250 mm) with a linear gradient of the binary solvent system of H_2_O/ACN/TFA (A: 95/5/0.1; B: 5/95/0.1) with a flow rate of 20 mL/min. Mass spectra were obtained with an Agilent 6410 Triple Quadrupole Mass Spectrometer instrument using electron spray coupled to an Agilent 1200 HPLC system (ESI-LC-MS) with a C18 reverse phase column (Zorbax Eclipse XBD-C18, 4.6 mm × 50 mm), autosampler and diode array detector using a linear gradient of the binary solvent system of H_2_O/ACN/formic acid (A: 95/5/0.1; B: 5/95/0.086) with a flow rate of 1 mL/min. During ESI-LC-MS analysis, evaporative light scattering (ELS) traces were obtained with a Sedere Sedex 85 Light Scattering Detector. Compound purity was confirmed by ESI-LC-MS to be >95% (UV and ELSD) for all tested compounds.

### 4-((3,5-Dichloro-2-hydroxybenzyl)amino)-2-hydroxybenzoic acid (ZL006)

ZL006 was purchased (Sigma-Aldrich) and purity checked by LC-MS to be >95%.

### 2-((1*H*-Benzo[d][1–3]triazol-5-ylamino)methyl)-4-6-dichlorophenol (IC87201)

5-Amino-1*H*-benzotriazole (0.210 g, 1.57 mmol, 1 equiv) and 3,5-dichloro-2-hydroxybenzaldehyde (0.300 g, 1.57 mmol, 1 equiv) were dissolved in toluene (15 mL) and stirred overnight under reflux. The solvent was removed *in vacuo,* and the resulting solid was dissolved in MeOH (15 mL) and NaBH_4_ (0.089 g, 2.36 mmol, 1.5 equiv) was added. The mixture was stirred at room temperature for 1 hour and the solvent was removed *in vacuo*. The resulting solid was dissolved in DCM, washed with H_2_O, dried with MgSO_4_, filtered, and concentrated to obtain a brown solid (0.170 g, 0.55 mmol, 35%). mp: 215 °C. TLC (SiO_2_, Cyc:EtOAc, 5/5 v/v): R_f_ = 0.17. ^1^H NMR (400 MHz, MeOD) δ 7.65 (d, *J* = 8.5, 1H, H_ar_), 7.23 (d, *J* = 2.5, 1H, H_ar_), 7.18 (d, *J* = 2.5, 1H, H_ar_), 6.92 (d, *J* = 8.5, 1H, H_ar_), 6.47 (s, 1H, H_ar_), 4.40 (s, 2H, CH_2_); ^13^C NMR (101 MHz, MeOD) δ 151.0 (C), 130.9 (3 C), 128.3 (2 CH), 127.6 (3 CH), 125.7 (2 C), 122.4 (C), 43.6 (CH_2_). HRMS (ESI+) (m/z): [M + H]^+^ calcd. for C_14_H_10_Cl_2_N_4_O, 309.0310; found 309.0305.

### Peptide Synthesis

Peptides were manually synthesized by Fmoc-based solid-phase peptide synthesis using a MiniBlock (Mettler-Toledo, Columbus, OH). 2-Chlorotrityl chloride polystyrene resins (1–2% DVB cross-linking, 100–200 mesh) pre-loaded with the first Fmoc-protected amino acid were used as solid supports. Fmoc deprotection was performed with 20% piperidine in DMF (1 × 5 and 1 × 15 min; wash with DMF in between), and coupling of the consecutive amino acid was carried out with *O*-(benzotriazol-1-yl)-*N*,*N*,*N*’,*N*’-tetramethyluronium hexafluorophosphate (HBTU) and DIPEA (peptide-resin/amino acid/HBTU/DIPEA 1:4:4:4 equiv) in dry DMF (~2 mL per 0.25 mmol resin) for 30 min. Final peptides were obtained by cleaving the resin-bound peptide with TFA/TIPS/H_2_O (90:5:5) for 2 hours followed by evaporation in vacuo, cold ether precipitation, lyophilization, and HPLC purification. Labelled peptides were synthesized by attaching 5(6)-carboxytetramethylrhodamine (TAMRA) (Sigma-Aldrich) to the *N*-terminal amino group of the resin-bound peptide by coupling with *O*-(7-azabenzotriazol-1-yl)-*N*,*N*,*N*’,*N*’-tetramethyluronium hexafluorophosphate (HATU) and 2,4,6-trimethylpyridine as base (peptide-resin/TAMRA/HATU/2,4,6-trimethylpyridine 1:2:2:3 equiv) for 24 hours in DMF; or alternatively, by coupling Cy5-maleimide (GE Healthcare, UK) to an *N*-terminal cysteine residue on the peptide in solution (peptide/Cy5-maleimide 1:1 equiv, 1 × TBS buffer [150 mM NaCl, 10 mM Tris, pH 7.4], 2 hours at room temperature). See Table S1 for a complete list of peptides and peptide-based compounds used and/or synthesized in this work.

### Protein Expression

PDZ1 (61-151), PDZ2 (155-249), PDZ3 (309-401), and PDZ1-2 (61-249) from PSD-95 (numbers in parenthesis refer to the residue numbers in the human full-length PSD-95α without exon 4b) were expressed in *E. coli* (BL21-DE3, pLysS) and purified using a nickel(II)-charged HisTrap column (GE Healthcare Life Sciences, Uppsala, Sweden) followed by anion-exchange chromatography or gel-filtration as described previously^16^. These PDZ constructs contain an N-terminal His-tag sequence, MHHHHHPRGS, to facilitate purification.

nNOS-PDZ (12–130, human) and α-Syntrophin-PDZ (82–200, human), both comprising an N-terminal His-tag/FXa-site sequence (MHHHHHHHGGIEGRKL), were likewise expressed in *E. coli* and purified by HisTrap chromatography followed by gel-filtration as described in details previously^19^. To make the TAMRA-labelled nNOS-PDZ, mutant nNOS-PDZ (V36C) protein was generated by standard site-directed mutagenesis and characterized as described previously^19^. Here, dithiothreitol was added to the pooled protein sample after HisTrap purification to a final concentration of 10 mM to eliminate unwanted disulfide bonds, and a final gel-filtration (50 mM NaPi buffer pH 7.5) was performed to remove impurities and salts. Next, purified nNOS-PDZ (V36C) was desalted by dialysis into 50 mM (NH_4_)_2_CO_3_ buffer (SnakeSkin dialysis tubing 3.5 MWCO, Pierce, Rockford, IL, USA) and subsequently lyophilized. nNOS-PDZ (V36C) was redissolved in 500 μL buffer (6 M GnHCl, 200 mM NaPi) and solid TAMRA-maleimide (5-tetramethylrhodamine-C2-maleimide; Anaspec, Fremont, CA, USA) was added in excess (cf. ESI-LC-MS). The coupling reaction was incubated at room temperature for 16 hours and TAMRA-labelled nNOS-PDZ was purified by preparative HPLC, and refolded in assay buffer. Functional validation was performed by FP^19^, and all final proteins were characterized for purity and identity by SDS-PAGE and ESI-LC-MS.

For isothermal titration calorimetry (ITC) and NMR, PSD-95-PDZ2 (157–249, human) was expressed and purified as described previously^37^. The sequence for the extended *Mus musculus* nNOS-PDZ (residues 12–134, Uniprot Q9JJ30) was sub-cloned into a pETM11 vector using cloning sites Nco1 and Kpn1, and the protein was expressed and purified following a similar protocol to that used for the production of PSD-95-PDZ2.

### Fluorescence Polarization Assay

FP was generally performed as previously described^11^,^16^,^32^,^33^, but with the following specifications: Saturation binding experiments were performed for measuring binding affinity (*K*_d_) between TAMRA- or Cy5-labelled fluorescent peptide or protein (i.e. the probe) and the PDZ domain protein by applying an increasing amount of PDZ domain protein (typically 0–150 μM) to a fixed and low concentration of probe (5 or 50 nM). Incubation time was 10–15 minutes (room temperature), and the assay was performed in a 1 × TBS buffer (150 mM NaCl, 10 mM Tris, pH 7.4) using black flat-bottom 384-well plates (Corning Life Sciences, NY) and a Safire2 plate-reader (Tecan, Männedorf, Switzerland). G-factor was adjusted so that probe alone (i.e. without protein interacting-partner) would give an FP value of 20 mP. This background value was subsequently subtracted from the raw data before plotting and analyzing the data. TAMRA- and Cy5-probes were measured at excitation/emission values of 530/585 nm (bandwidth = 20 nm) and 635/670 nm (bandwidth = 15 nm), respectively. The FP values were fitted to the equation Y = B_max_ × X/(*K*_d_ + X), with B_max_ being the maximal FP value, X is the PDZ concentration, and Y is the experimental FP values. As long as the concentration of labelled peptide is well below the true *K*_d_ during the assay, the *K*_d_ can be directly derived from this saturation curve as being equal to the PDZ concentration where the curve is half-saturated (at these conditions EC_50 _ ≡ [total PDZ]_half-saturation_ = [free PDZ]_half-saturation_ ∫ *K*_d_)^42^.

To measure inhibition of probe and protein by test substance (controls compounds, ZL006, IC87201, or unlabelled protein or peptide), heterologous competition binding assays were performed by adding increasing concentrations of test substance to a fixed concentration of probe and protein under the same conditions as in the saturation binding experiments. FP values were fitted to the general equation: Y = Bottom + (Top–Bottom)/[1 + (10^(X-logIC50)*HillSlope^)], where X is the logarithmic value of peptide/compound concentration, and Y is the experimental FP values. Competitive inhibition constants, *K*_i_ values, were calculated from the IC_50_ values to quantify affinities between test substance and protein^42^.

To measure the expected ability of ZL006 and IC87201 to inhibit the PSD-95-PDZ/nNOS-PDZ interaction using unlabelled protein, an indirect FP experiment was established using a probe (Cy5/TAMRA-Cnksr) that selectively binds PSD-95-PDZ2 (or PDZ1-2) but not nNOS-PDZ (Fig. S1, Fig. 4a). Increasing concentrations of ZL006 or IC87201 were added to a fixed concentration of PSD-95-PDZ, nNOS-PDZ, and probe (Fig. 4c–d). If IC87201 and ZL006 bind nNOS-PDZ and inhibit the PSD-95-PDZ/nNOS-PDZ interaction, PSD-95-PDZ would get liberated from the interaction and instead bind to the probe and thus give rise to an increase in FP signal in accordance with the inhibition curve of nNOS vs PDZ/Cnskr (Fig. 4b). Similar, this assay was established with Syntrophin-PDZ and Sapk3 as probe (Fig. 4d).

ZL006 and IC87201 stocks were prepared in 100% DMSO and dilution curves were generated using 1 × TBS buffer and, if necessary to get full solubilisation, extra DMSO. Final DMSO concentrations in the FP assay did not exceed 10% for IC87201 and 20% for ZL006 at their highest test concentrations; and in test concentrations ≤600 μM, DMSO concentrations did not exceed 5% and dropped proportionally with compound concentrations. In all cases, the DMSO effect on the assay was tested and found negligible.

### Isothermal Titration Calorimetry

All ITC experiments were carried out at 25 °C on an iTC200 Microcalorimeter (GE Healthcare) with a 200 μL cell capacity and 40 μL syringe volume. The buffer used for all solutions was 20 mM phosphate buffer at pH 6.3; each experiment consisted of an initial injection of 0.5 μL, followed by fifteen 2.39 μL injections before a final injection of 1.89 μL. Control experiments were performed whereby IC87201 or ZL006 was titrated into buffer and buffer titrated into nNOS or PSD-95-PDZ2. In both cases no heat exchange was detected, confirming that there was appropriate match of buffer conditions with no indication of dilution effects. The ITC titration experiments for the interactions between IC87201 or ZL006 and each PDZ domain were performed in duplicates, with 100 μM nNOS-PDZ or PSD-95-PDZ2 in the cell and 750 μM IC87201 or ZL006 in the syringe, with 1% DMSO present in all the solutions. For the nNOS-PDZ/PSD-95-PDZ2 interaction, 100 μM PDZ2 in the cell was titrated with 750 μM nNOS-PDZ. To investigate whether IC87201 or ZL006 was able to inhibit the nNOS-PDZ/PSD-95-PDZ2 interaction, the ITC titration was performed as in the nNOS-PDZ/PSD-95-PDZ2 interaction but with 1 mM IC87201 or ZL006 (i.e. PSD-95 PDZ2:IC87201 or ZL006 = 1:10) present in both the PSD-95-PDZ2 and the nNOS-PDZ samples. All data was analysed using the Origin®7 software programme.

### ^1^H-^15^N HSQC NMR

The NMR spectra were acquired at 25 °C on Bruker AVANCE II 600 MHz or 800 MHz spectrometers, equipped with triple resonance cryoprobes. The Bruker TopSpin programme version 3.1 was used to process the resultant NMR spectra and the Collaborative Computational Project for NMR (CCPN) Analysis software programme was used for interactive spectral analysis. 2-D ^1^H-^15^N HSQC NMR experiments^43^ were performed using 0.05–0.25 mM ^15^N-labelled nNOS-PDZ or PSD-95-PDZ2 in 20 mM phosphate buffered to pH 6.3. When required, IC87201 or ZL006 in DMSO-d_6_ was added to a 10-20-fold excess relative to protein.

## Additional Information

**How to cite this article**: Bach, A. *et al.* Biochemical investigations of the mechanism of action of small molecules ZL006 and IC87201 as potential inhibitors of the nNOS-PDZ/PSD-95-PDZ interactions. *Sci. Rep.* **5**, 12157; doi: 10.1038/srep12157 (2015).

## Supplementary Material

Supporting Information

## Figures and Tables

**Figure 1:**
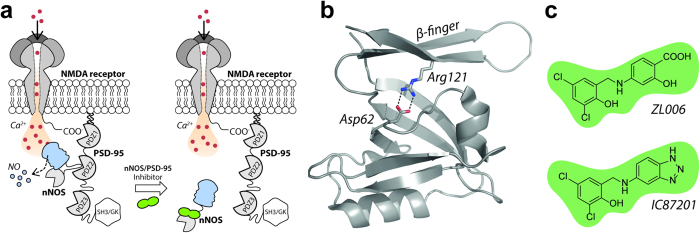
Mechanism of action under investigation. (**a**) The neuronal nNOS/PSD-95/NMDA receptor complex and proposed mechanism of nNOS/PSD-95 inhibitors. (**b**) The extended nNOS-PDZ domain including the β-finger, which is here studied for engagement with ZL006 and IC87201. (**c**) Chemical structures of ZL006 and IC87201.
