# Supplementary material for: Biochemical investigations of the mechanism of action of small molecules ZL006 and IC87201 as potential inhibitors of the nNOS-PDZ/PSD-95-PDZ interactions
Source: Sci Rep. 2015 Jul 16;5:12157. doi: 10.1038/srep12157 (PMC4503980; doi:10.1038/srep12157)
Supplement: Supporting Information [file srep12157-s1.pdf]

Biochemical investigations of the mechanism of action of small molecules ZL006 and IC87201 as potential inhibitors of the nNOS-PDZ/PSD-95-PDZ interactions.

Anders Bach[1,*], Søren W. Pedersen[1], Liam A. Dorr[2], Gary Vallon[3], Isabelle Ripoche[3], Sylvie Ducki[3], and Lu-Yun Lian[2,*]

[1] Department of Drug Design and Pharmacology, Faculty of Health and Medical Sciences, University of Copenhagen, Universitetsparken 2, DK-2100 Copenhagen, Denmark.

[2] NMR Centre for Structural Biology, University of Liverpool, UK L69 7ZB Liverpool

[3] Clermont Université, ENSCCF, CNRS UMR 6296, Institut de Chimie de Clermont-Ferrand, BP10187, F-63174 Aubière, France

SUPPLEMENTARY INFORMATION

Supplementary Figure S1

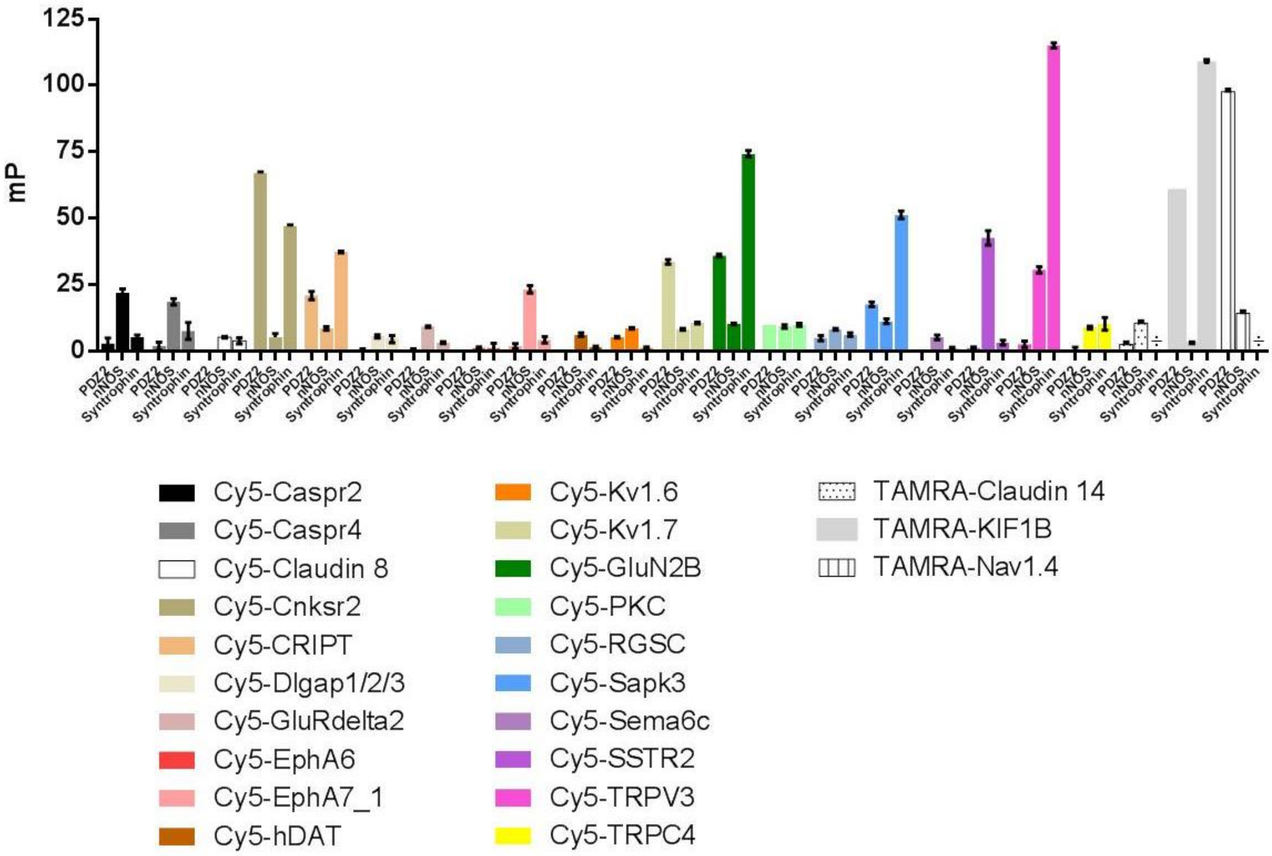

Figure S1. Screening for selective probes. An array of fluorescent peptides (Cy5- or TAMRA-probes) known to bind various PDZ domains[1] at 5 nM were screened by FP against PSD-95-PDZ2 (5 µM), nNOS-PDZ (5 µM), and Syntrophin-PDZ (5 µM), to find the most selective probes. Cnksr2 showed the desired selectivity of PDZ2 over nNOS; and Sapk3 showed selectivity of Syntrophin over nNOS. Error bars indicate SEM based on three measurements. ÷: Not tested.

Supplementary Table S1. Peptides and peptide-based compounds synthesized and/or used.

Compound	Sequence/Structure[a]	M_w[b]	Purity[c]
Cy5-GluN2B	Cy5-CSG-YEKLSSIESDV	(Bach et al, 2008[2])	
Cy5-CRIPT	Cy5-CSG-LDTKNYKQTSV	(Bach et al, 2008[2])	
Cy5-Caspr2	Cy5-CNNG-IDESKKEWLI	2426.7	>95%
Cy5-Caspr4	Cy5-CNNG-VGENQKEYFF	2426.7	>95%
Cy5-Claudin 8	Cy5-CNNG-PSIYSKSQYV	2337.6	>95%
Cy5-Claudin 14	Cy5-CNNG-HSGYRLNDYV	2389.6	>95%
Cy5-Cnksr2	Cy5-CNNG-HTHSYIETHV	2389.6	>95%
Cy5-Dlgap1/2/3	Cy5-CNNG-IYIPEAQTRL	2369.7	>95%
Cy5-GluRdelta2	Cy5-CNNG-GNDPDRGTSI	2197.3	>95%
Cy5-EphA6	Cy5-CNNG-MHIQEKGFHV	2391.7	>95%
Cy5-EphA7_1	Cy5-CNNG-LHLHGTGIQV	2240.5	>95%
Cy5-hDAT	Cy5-CNNG-QFTLRHWLKV	2493.9	>95%
Cy5-KIF1B	Cy5-CNNG-NLKAGRETTV	2254.5	>95%
Cy5-Kv1.6	Cy5-CNNG-YAEKRMLTEV	2405.7	>95%
Cy5-Kv1.7	Cy5-CNNG-PAGKHMVTEV	2234.5	>95%
Cy5-Nav1.4	Cy5-CNNG-VRPGVKESLV	2249.6	>95%
Cy5-PKC	Cy5-CNNG-FVHPILQSAV	2276.6	>95%
Cy5-RGSC	Cy5-CNNG-KTSAHHATFV	2263.9	>95%
Cy5-Sapk3	Cy5-CNNG-GARVPKETAL	2207.5	>95%
Cy5-Sema6c	Cy5-CNNG-PAPHGGHFNF	2246.5	>95%
Cy5-SSTR2	Cy5-CNNG-SGAEDIIAWV	2226.5	>95%
Cy5-TRPV3	Cy5-CNNG-ELDEFPETSV	2331.5	>95%
Cy5-TRPC4	Cy5-CNNG-AHEDYVTTRL	2370.6	>95%
TAMRA-Cnksr2	TAMRA-NNG-HTHSYIETHV	1922.0	>95%
Nav1.4-C10	NNG-VRPGVKESLV	1368.6	>95%
GluN2B-C5	IESDV	(Bach et al, 2008[2])	
CRIPT-C11	KSG-LDTKNYKQTSV	(Bach et al, 2008[2])	
N-Cyclohexylethyl-ETAV	*N*-Cyclohexylethyl-ETAV	(Bach et al, 2008 and 2011[2,3])	
Tat-*N*-dimer	Tat-*N*PEG4$(IETDV)_2$	(Bach et al, 2012[4])	

[a] CSG, KSG, NNG, and CNNG are tri- or tetrapeptide linkers introduced between the fluorophore (Cy5 or TAMRA) and the PDZ ligand-sequence (See also references[1,2])

[b] Calculated molecular weight (Da)

[c] Purity determined by ESI-LC-MS

References

1 Stiffler, M. A. *et al.* PDZ domain binding selectivity is optimized across the mouse proteome. *Science* **317**, 364-369 (2007).

2 Bach, A. *et al.* Modified peptides as potent inhibitors of the postsynaptic density-95/*N*-methyl-D-aspartate receptor interaction. *J. Med. Chem.* **51**, 6450-6459 (2008).

3 Bach, A. *et al.* Cell-permeable and plasma-stable peptidomimetic inhibitors of the postsynaptic density-95/*N*-methyl-D-aspartate receptor interaction. *J. Med. Chem.* **54**, 1333-1346 (2011).

4 Bach, A. *et al.* A high-affinity, dimeric inhibitor of PSD-95 bivalently interacts with PDZ1-2 and protects against ischemic brain damage. *Proc. Natl. Acad. Sci. U. S. A.* **109**, 3317-3322 (2012).
